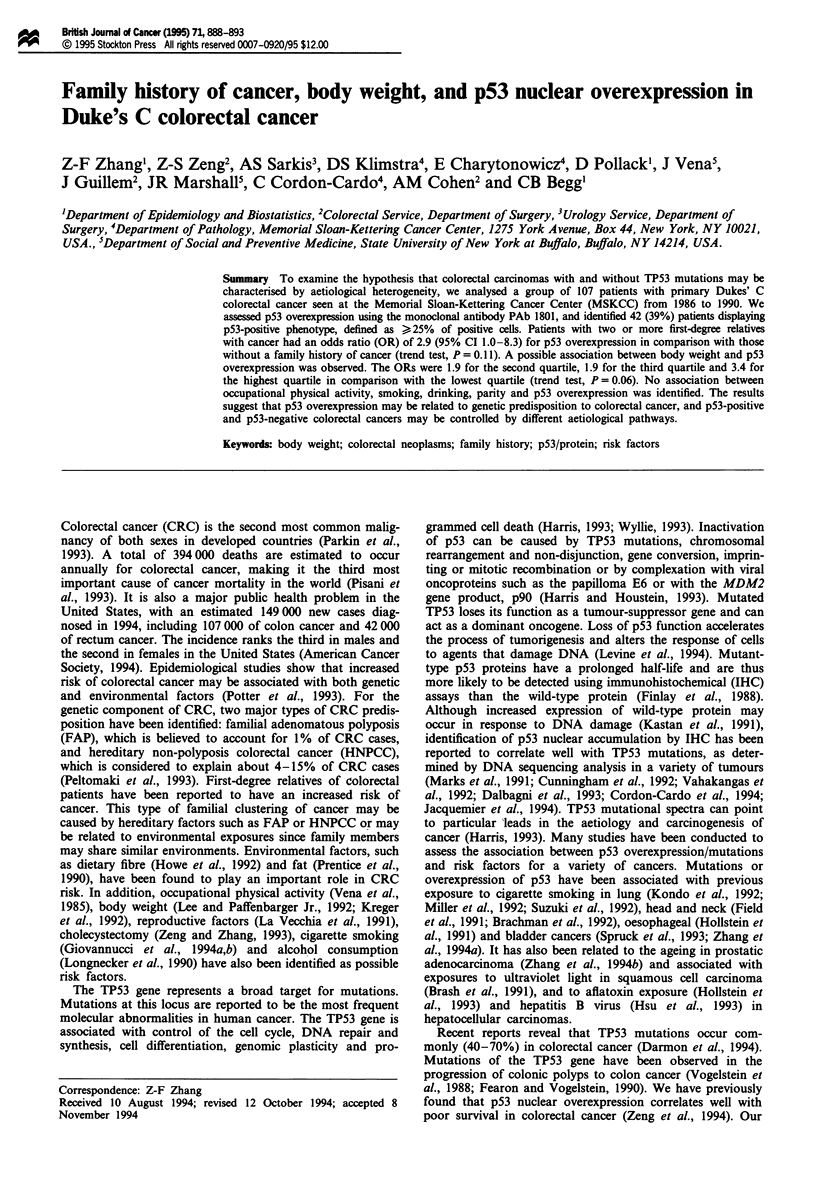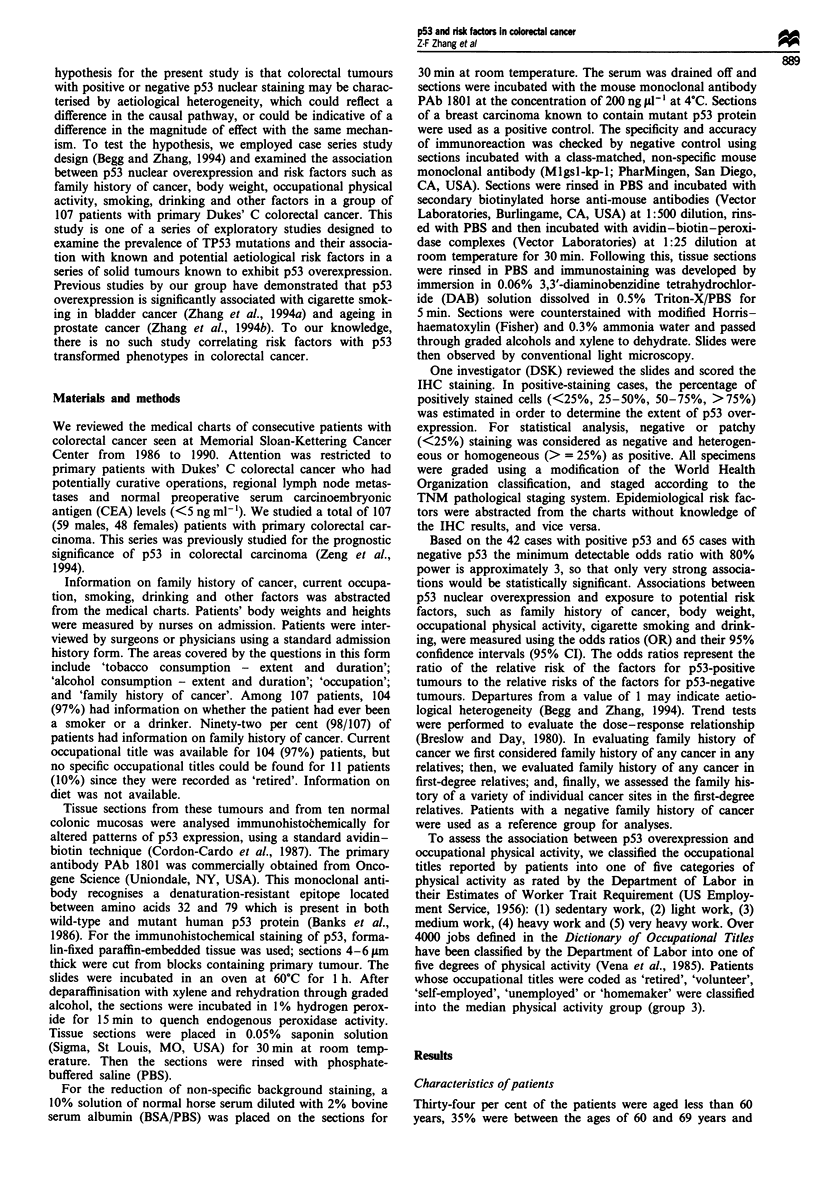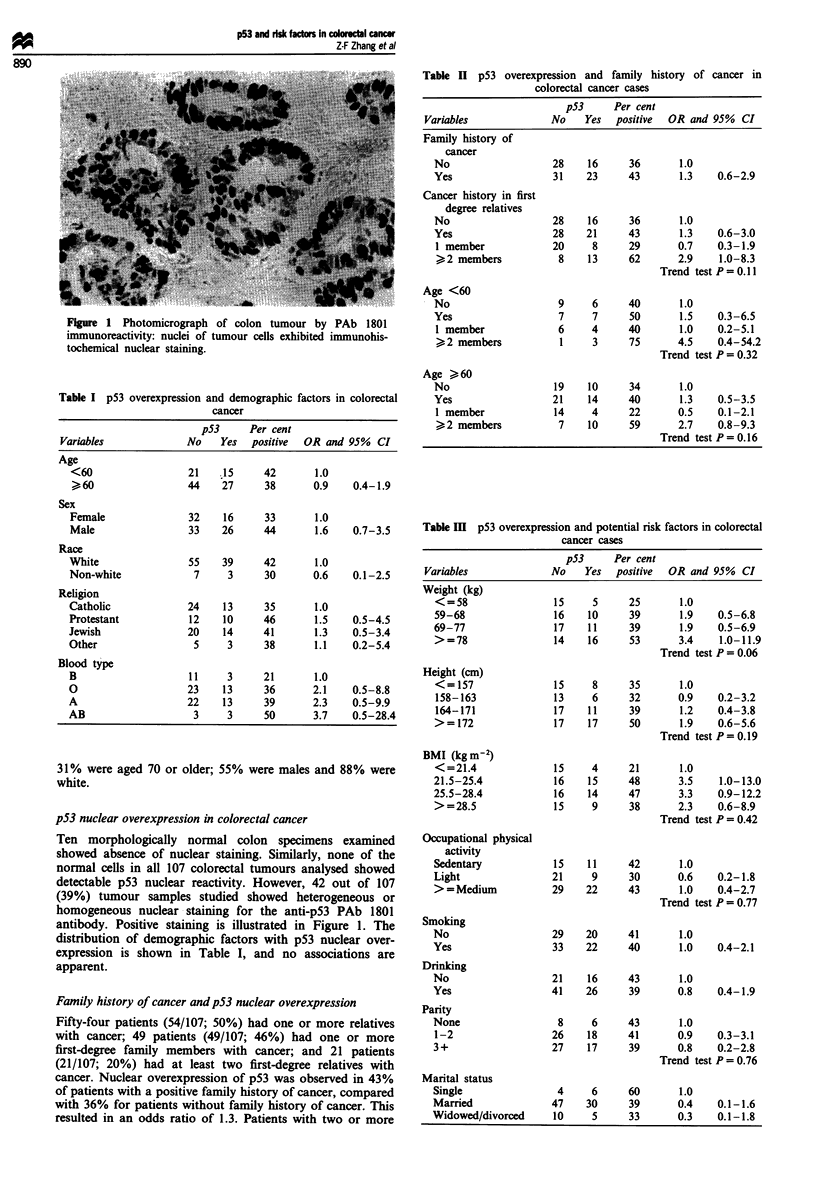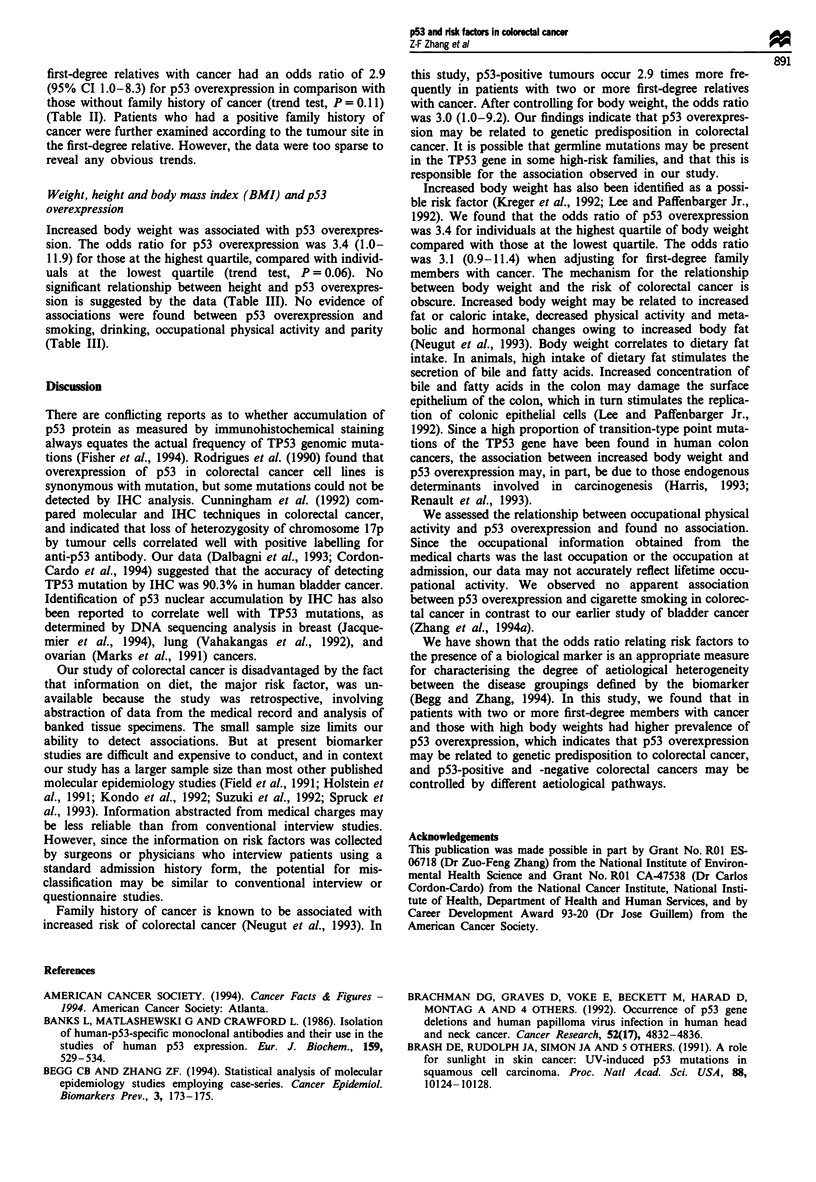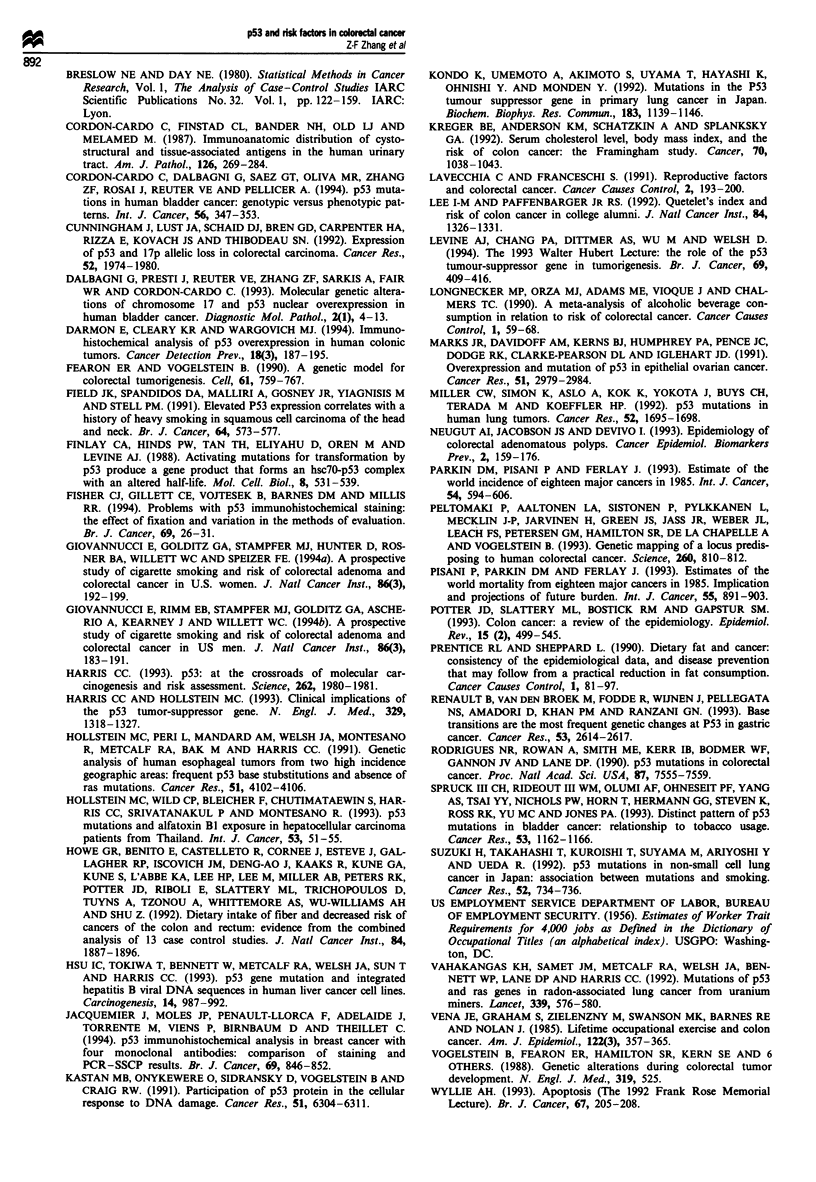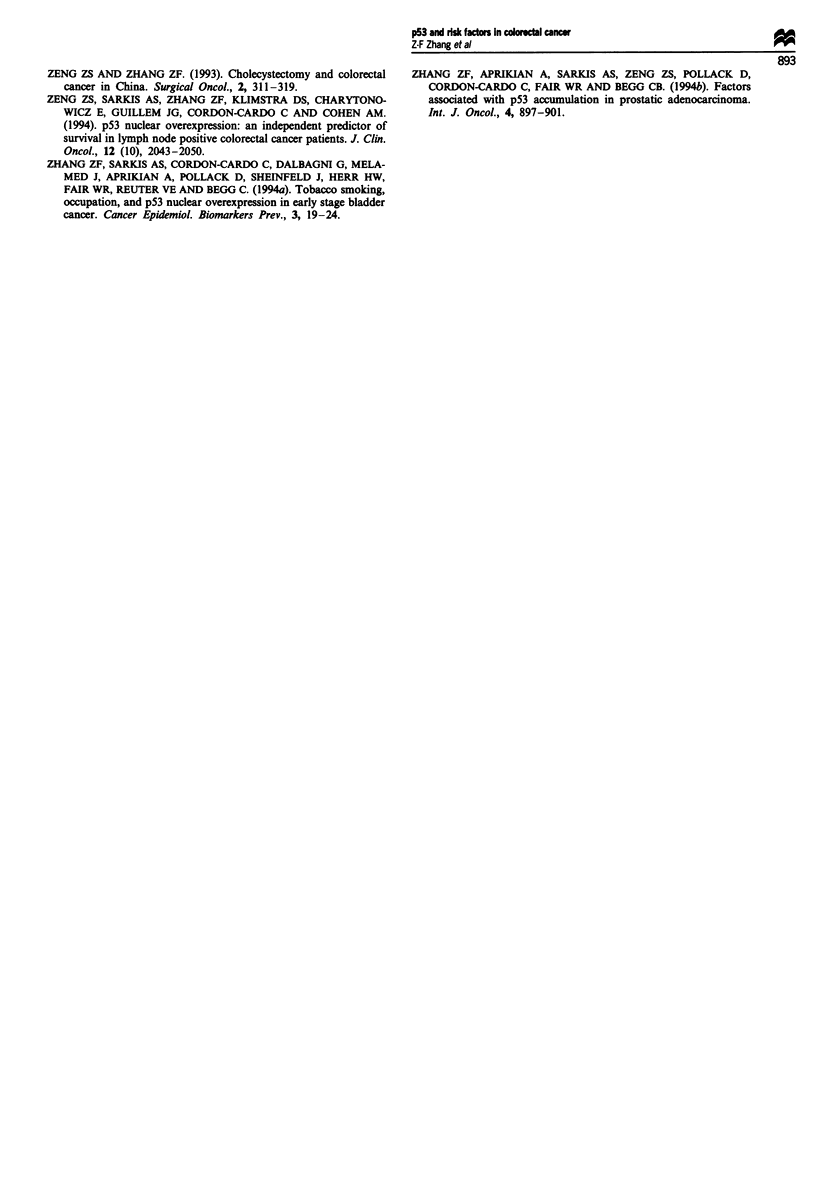# Family history of cancer, body weight, and p53 nuclear overexpression in Duke's C colorectal cancer.

**DOI:** 10.1038/bjc.1995.171

**Published:** 1995-04

**Authors:** Z. F. Zhang, Z. S. Zeng, A. S. Sarkis, D. S. Klimstra, E. Charytonowicz, D. Pollack, J. Vena, J. Guillem, J. R. Marshall, C. Cordon-Cardo

**Affiliations:** Department of Epidemiology and Biostatistics, Memorial Sloan-Kettering Cancer Center, New York, NY 10021, USA.

## Abstract

**Images:**


British Journal of Cancer (1995) 71, 888-893
© 1995 Stockton Press All rights reserved 0007-0920/95 $12.00

### Family history of cancer, body weight, and p53 nuclear overexpression in Duke's C colorectal cancer

Z-F Zhang[1], Z-S Zeng[2], AS Sarkis[3], DS Klimstra[4], E Charytonowicz[4], D Pollack[1], J Vena[5], J Guillem[2], JR Marshall[5], C Cordon-Cardo[4], AM Cohen[2] and CB Begg[1]

[1]*Department of Epidemiology and Biostatistics,* [2]*Colorectal Service, Department of Surgery,* [3]*Urology Service, Department of Surgery,* [4]*Department of Pathology, Memorial Sloan-Kettering Cancer Center, 1275 York Avenue, Box 44, New York, NY 10021, USA.,* [5]*Department of Social and Preventive Medicine, State University of New York at Buffalo, Buffalo, NY 14214, USA.*

**Summary** To examine the hypothesis that colorectal carcinomas with and without TP53 mutations may be characterised by aetiological heterogeneity, we analysed a group of 107 patients with primary Dukes' C colorectal cancer seen at the Memorial Sloan-Kettering Cancer Center (MSKCC) from 1986 to 1990. We assessed p53 overexpression using the monoclonal antibody PAb 1801, and identified 42 (39%) patients displaying p53-positive phenotype, defined as $\geqslant 25\%$ of positive cells. Patients with two or more first-degree relatives with cancer had an odds ratio (OR) of 2.9 (95% CI 1.0-8.3) for p53 overexpression in comparison with those without a family history of cancer (trend test, $P = 0.11$). A possible association between body weight and p53 overexpression was observed. The ORs were 1.9 for the second quartile, 1.9 for the third quartile and 3.4 for the highest quartile in comparison with the lowest quartile (trend test, $P = 0.06$). No association between occupational physical activity, smoking, drinking, parity and p53 overexpression was identified. The results suggest that p53 overexpression may be related to genetic predisposition to colorectal cancer, and p53-positive and p53-negative colorectal cancers may be controlled by different aetiological pathways.

**Keywords:** body weight; colorectal neoplasms; family history; p53/protein; risk factors

Colorectal cancer (CRC) is the second most common malignancy of both sexes in developed countries (Parkin *et al.*, 1993). A total of 394 000 deaths are estimated to occur annually for colorectal cancer, making it the third most important cause of cancer mortality in the world (Pisani *et al.*, 1993). It is also a major public health problem in the United States, with an estimated 149 000 new cases diagnosed in 1994, including 107 000 of colon cancer and 42 000 of rectum cancer. The incidence ranks the third in males and the second in females in the United States (American Cancer Society, 1994). Epidemiological studies show that increased risk of colorectal cancer may be associated with both genetic and environmental factors (Potter *et al.*, 1993). For the genetic component of CRC, two major types of CRC predisposition have been identified: familial adenomatous polyposis (FAP), which is believed to account for 1% of CRC cases, and hereditary non-polyposis colorectal cancer (HNPCC), which is considered to explain about 4-15% of CRC cases (Peltomaki *et al.*, 1993). First-degree relatives of colorectal patients have been reported to have an increased risk of cancer. This type of familial clustering of cancer may be caused by hereditary factors such as FAP or HNPCC or may be related to environmental exposures since family members may share similar environments. Environmental factors, such as dietary fibre (Howe *et al.*, 1992) and fat (Prentice *et al.*, 1990), have been found to play an important role in CRC risk. In addition, occupational physical activity (Vena *et al.*, 1985), body weight (Lee and Paffenbarger Jr., 1992; Kreger *et al.*, 1992), reproductive factors (La Vecchia *et al.*, 1991), cholecystectomy (Zeng and Zhang, 1993), cigarette smoking (Giovannucci *et al.*, 1994*a,b*) and alcohol consumption (Longnecker *et al.*, 1990) have also been identified as possible risk factors.

The TP53 gene represents a broad target for mutations. Mutations at this locus are reported to be the most frequent molecular abnormalities in human cancer. The TP53 gene is associated with control of the cell cycle, DNA repair and synthesis, cell differentiation, genomic plasticity and programmed cell death (Harris, 1993; Wyllie, 1993). Inactivation of p53 can be caused by TP53 mutations, chromosomal rearrangement and non-disjunction, gene conversion, imprinting or mitotic recombination or by complexation with viral oncoproteins such as the papilloma E6 or with the *MDM2* gene product, p90 (Harris and Houstein, 1993). Mutated TP53 loses its function as a tumour-suppressor gene and can act as a dominant oncogene. Loss of p53 function accelerates the process of tumorigenesis and alters the response of cells to agents that damage DNA (Levine *et al.*, 1994). Mutant-type p53 proteins have a prolonged half-life and are thus more likely to be detected using immunohistochemical (IHC) assays than the wild-type protein (Finlay *et al.*, 1988). Although increased expression of wild-type protein may occur in response to DNA damage (Kastan *et al.*, 1991), identification of p53 nuclear accumulation by IHC has been reported to correlate well with TP53 mutations, as determined by DNA sequencing analysis in a variety of tumours (Marks *et al.*, 1991; Cunningham *et al.*, 1992; Vahakangas *et al.*, 1992; Dalbagni *et al.*, 1993; Cordon-Cardo *et al.*, 1994; Jacquemier *et al.*, 1994). TP53 mutational spectra can point to particular leads in the aetiology and carcinogenesis of cancer (Harris, 1993). Many studies have been conducted to assess the association between p53 overexpression/mutations and risk factors for a variety of cancers. Mutations or overexpression of p53 have been associated with previous exposure to cigarette smoking in lung (Kondo *et al.*, 1992; Miller *et al.*, 1992; Suzuki *et al.*, 1992), head and neck (Field *et al.*, 1991; Brachman *et al.*, 1992), oesophageal (Hollstein *et al.*, 1991) and bladder cancers (Spruck *et al.*, 1993; Zhang *et al.*, 1994*a*). It has also been related to the ageing in prostatic adenocarcinoma (Zhang *et al.*, 1994*b*) and associated with exposures to ultraviolet light in squamous cell carcinoma (Brash *et al.*, 1991), and to aflatoxin exposure (Hollstein *et al.*, 1993) and hepatitis B virus (Hsu *et al.*, 1993) in hepatocellular carcinomas.

Recent reports reveal that TP53 mutations occur commonly (40-70%) in colorectal cancer (Darmon *et al.*, 1994). Mutations of the TP53 gene have been observed in the progression of colonic polyps to colon cancer (Vogelstein *et al.*, 1988; Fearon and Vogelstein, 1990). We have previously found that p53 nuclear overexpression correlates well with poor survival in colorectal cancer (Zeng *et al.*, 1994). Our

Correspondence: Z-F Zhang
Received 10 August 1994; revised 12 October 1994; accepted 8 November 1994

hypothesis for the present study is that colorectal tumours with positive or negative p53 nuclear staining may be characterised by aetiological heterogeneity, which could reflect a difference in the causal pathway, or could be indicative of a difference in the magnitude of effect with the same mechanism. To test the hypothesis, we employed case series study design (Begg and Zhang, 1994) and examined the association between p53 nuclear overexpression and risk factors such as family history of cancer, body weight, occupational physical activity, smoking, drinking and other factors in a group of 107 patients with primary Dukes' C colorectal cancer. This study is one of a series of exploratory studies designed to examine the prevalence of TP53 mutations and their association with known and potential aetiological risk factors in a series of solid tumours known to exhibit p53 overexpression. Previous studies by our group have demonstrated that p53 overexpression is significantly associated with cigarette smoking in bladder cancer (Zhang *et al.*, 1994*a*) and ageing in prostate cancer (Zhang *et al.*, 1994*b*). To our knowledge, there is no such study correlating risk factors with p53 transformed phenotypes in colorectal cancer.

### Materials and methods

We reviewed the medical charts of consecutive patients with colorectal cancer seen at Memorial Sloan-Kettering Cancer Center from 1986 to 1990. Attention was restricted to primary patients with Dukes' C colorectal cancer who had potentially curative operations, regional lymph node metastases and normal preoperative serum carcinoembryonic antigen (CEA) levels ($<5$ ng ml$^{-1}$). We studied a total of 107 (59 males, 48 females) patients with primary colorectal carcinoma. This series was previously studied for the prognostic significance of p53 in colorectal carcinoma (Zeng *et al.*, 1994).

Information on family history of cancer, current occupation, smoking, drinking and other factors was abstracted from the medical charts. Patients' body weights and heights were measured by nurses on admission. Patients were interviewed by surgeons or physicians using a standard admission history form. The areas covered by the questions in this form include 'tobacco consumption - extent and duration'; 'alcohol consumption - extent and duration'; 'occupation'; and 'family history of cancer'. Among 107 patients, 104 (97%) had information on whether the patient had ever been a smoker or a drinker. Ninety-two per cent (98/107) of patients had information on family history of cancer. Current occupational title was available for 104 (97%) patients, but no specific occupational titles could be found for 11 patients (10%) since they were recorded as 'retired'. Information on diet was not available.

Tissue sections from these tumours and from ten normal colonic mucosas were analysed immunohistochemically for altered patterns of p53 expression, using a standard avidin–biotin technique (Cordon-Cardo *et al.*, 1987). The primary antibody PAb 1801 was commercially obtained from Oncogene Science (Uniondale, NY, USA). This monoclonal antibody recognises a denaturation-resistant epitope located between amino acids 32 and 79 which is present in both wild-type and mutant human p53 protein (Banks *et al.*, 1986). For the immunohistochemical staining of p53, formalin-fixed paraffin-embedded tissue was used; sections 4–6 μm thick were cut from blocks containing primary tumour. The slides were incubated in an oven at 60°C for 1 h. After deparaffinisation with xylene and rehydration through graded alcohol, the sections were incubated in 1% hydrogen peroxide for 15 min to quench endogenous peroxidase activity. Tissue sections were placed in 0.05% saponin solution (Sigma, St Louis, MO, USA) for 30 min at room temperature. Then the sections were rinsed with phosphate-buffered saline (PBS).

For the reduction of non-specific background staining, a 10% solution of normal horse serum diluted with 2% bovine serum albumin (BSA/PBS) was placed on the sections for 30 min at room temperature. The serum was drained off and sections were incubated with the mouse monoclonal antibody PAb 1801 at the concentration of 200 ng μl$^{-1}$ at 4°C. Sections of a breast carcinoma known to contain mutant p53 protein were used as a positive control. The specificity and accuracy of immunoreaction was checked by negative control using sections incubated with a class-matched, non-specific mouse monoclonal antibody (M1gs1-kp-1; PharMingen, San Diego, CA, USA). Sections were rinsed in PBS and incubated with secondary biotinylated horse anti-mouse antibodies (Vector Laboratories, Burlingame, CA, USA) at 1:500 dilution, rinsed with PBS and then incubated with avidin–biotin–peroxidase complexes (Vector Laboratories) at 1:25 dilution at room temperature for 30 min. Following this, tissue sections were rinsed in PBS and immunostaining was developed by immersion in 0.06% 3,3'-diaminobenzidine tetrahydrochloride (DAB) solution dissolved in 0.5% Triton-X/PBS for 5 min. Sections were counterstained with modified Horris–haematoxylin (Fisher) and 0.3% ammonia water and passed through graded alcohols and xylene to dehydrate. Slides were then observed by conventional light microscopy.

One investigator (DSK) reviewed the slides and scored the IHC staining. In positive-staining cases, the percentage of positively stained cells ($<25\%$, 25–50%, 50–75%, $>75\%$) was estimated in order to determine the extent of p53 overexpression. For statistical analysis, negative or patchy ($<25\%$) staining was considered as negative and heterogeneous or homogeneous ($>=25\%$) as positive. All specimens were graded using a modification of the World Health Organization classification, and staged according to the TNM pathological staging system. Epidemiological risk factors were abstracted from the charts without knowledge of the IHC results, and vice versa.

Based on the 42 cases with positive p53 and 65 cases with negative p53 the minimum detectable odds ratio with 80% power is approximately 3, so that only very strong associations would be statistically significant. Associations between p53 nuclear overexpression and exposure to potential risk factors, such as family history of cancer, body weight, occupational physical activity, cigarette smoking and drinking, were measured using the odds ratios (OR) and their 95% confidence intervals (95% CI). The odds ratios represent the ratio of the relative risk of the factors for p53-positive tumours to the relative risks of the factors for p53-negative tumours. Departures from a value of 1 may indicate aetiological heterogeneity (Begg and Zhang, 1994). Trend tests were performed to evaluate the dose–response relationship (Breslow and Day, 1980). In evaluating family history of cancer we first considered family history of any cancer in any relatives; then, we evaluated family history of any cancer in first-degree relatives; and, finally, we assessed the family history of a variety of individual cancer sites in the first-degree relatives. Patients with a negative family history of cancer were used as a reference group for analyses.

To assess the association between p53 overexpression and occupational physical activity, we classified the occupational titles reported by patients into one of five categories of physical activity as rated by the Department of Labor in their Estimates of Worker Trait Requirement (US Employment Service, 1956): (1) sedentary work, (2) light work, (3) medium work, (4) heavy work and (5) very heavy work. Over 4000 jobs defined in the *Dictionary of Occupational Titles* have been classified by the Department of Labor into one of five degrees of physical activity (Vena *et al.*, 1985). Patients whose occupational titles were coded as 'retired', 'volunteer', 'self-employed', 'unemployed' or 'homemaker' were classified into the median physical activity group (group 3).

### Results

### *Characteristics of patients*

Thirty-four per cent of the patients were aged less than 60 years, 35% were between the ages of 60 and 69 years and

**Figure 1** Photomicrograph of colon tumour by PAb 1801 immunoreactivity: nuclei of tumour cells exhibited immunohistochemical nuclear staining.

**Table I** p53 overexpression and demographic factors in colorectal cancer

| Variables | p53 No | p53 Yes | Per cent positive | OR and 95% CI | |
|---|---|---|---|---|---|
| Age | | | | | |
| <60 | 21 | 15 | 42 | 1.0 | |
| ≥60 | 44 | 27 | 38 | 0.9 | 0.4–1.9 |
| Sex | | | | | |
| Female | 32 | 16 | 33 | 1.0 | |
| Male | 33 | 26 | 44 | 1.6 | 0.7–3.5 |
| Race | | | | | |
| White | 55 | 39 | 42 | 1.0 | |
| Non-white | 7 | 3 | 30 | 0.6 | 0.1–2.5 |
| Religion | | | | | |
| Catholic | 24 | 13 | 35 | 1.0 | |
| Protestant | 12 | 10 | 46 | 1.5 | 0.5–4.5 |
| Jewish | 20 | 14 | 41 | 1.3 | 0.5–3.4 |
| Other | 5 | 3 | 38 | 1.1 | 0.2–5.4 |
| Blood type | | | | | |
| B | 11 | 3 | 21 | 1.0 | |
| O | 23 | 13 | 36 | 2.1 | 0.5–8.8 |
| A | 22 | 13 | 39 | 2.3 | 0.5–9.9 |
| AB | 3 | 3 | 50 | 3.7 | 0.5–28.4 |

31% were aged 70 or older; 55% were males and 88% were white.

### *p53 nuclear overexpression in colorectal cancer*

Ten morphologically normal colon specimens examined showed absence of nuclear staining. Similarly, none of the normal cells in all 107 colorectal tumours analysed showed detectable p53 nuclear reactivity. However, 42 out of 107 (39%) tumour samples studied showed heterogeneous or homogeneous nuclear staining for the anti-p53 PAb 1801 antibody. Positive staining is illustrated in Figure 1. The distribution of demographic factors with p53 nuclear overexpression is shown in Table I, and no associations are apparent.

### *Family history of cancer and p53 nuclear overexpression*

Fifty-four patients (54/107; 50%) had one or more relatives with cancer; 49 patients (49/107; 46%) had one or more first-degree family members with cancer; and 21 patients (21/107; 20%) had at least two first-degree relatives with cancer. Nuclear overexpression of p53 was observed in 43% of patients with a positive family history of cancer, compared with 36% for patients without family history of cancer. This resulted in an odds ratio of 1.3. Patients with two or more

**Table II** p53 overexpression and family history of cancer in colorectal cancer cases

| Variables | p53 No | p53 Yes | Per cent positive | OR and 95% CI | |
|---|---|---|---|---|---|
| Family history of cancer | | | | | |
| No | 28 | 16 | 36 | 1.0 | |
| Yes | 31 | 23 | 43 | 1.3 | 0.6–2.9 |
| Cancer history in first degree relatives | | | | | |
| No | 28 | 16 | 36 | 1.0 | |
| Yes | 28 | 21 | 43 | 1.3 | 0.6–3.0 |
| 1 member | 20 | 8 | 29 | 0.7 | 0.3–1.9 |
| ≥2 members | 8 | 13 | 62 | 2.9 | 1.0–8.3 |
| | | | | Trend test $P = 0.11$ | |
| Age <60 | | | | | |
| No | 9 | 6 | 40 | 1.0 | |
| Yes | 7 | 7 | 50 | 1.5 | 0.3–6.5 |
| 1 member | 6 | 4 | 40 | 1.0 | 0.2–5.1 |
| ≥2 members | 1 | 3 | 75 | 4.5 | 0.4–54.2 |
| | | | | Trend test $P = 0.32$ | |
| Age ≥60 | | | | | |
| No | 19 | 10 | 34 | 1.0 | |
| Yes | 21 | 14 | 40 | 1.3 | 0.5–3.5 |
| 1 member | 14 | 4 | 22 | 0.5 | 0.1–2.1 |
| ≥2 members | 7 | 10 | 59 | 2.7 | 0.8–9.3 |
| | | | | Trend test $P = 0.16$ | |

**Table III** p53 overexpression and potential risk factors in colorectal cancer cases

| Variables | p53 No | p53 Yes | Per cent positive | OR and 95% CI | |
|---|---|---|---|---|---|
| Weight (kg) | | | | | |
| <=58 | 15 | 5 | 25 | 1.0 | |
| 59–68 | 16 | 10 | 39 | 1.9 | 0.5–6.8 |
| 69–77 | 17 | 11 | 39 | 1.9 | 0.5–6.9 |
| >=78 | 14 | 16 | 53 | 3.4 | 1.0–11.9 |
| | | | | Trend test $P = 0.06$ | |
| Height (cm) | | | | | |
| <=157 | 15 | 8 | 35 | 1.0 | |
| 158–163 | 13 | 6 | 32 | 0.9 | 0.2–3.2 |
| 164–171 | 17 | 11 | 39 | 1.2 | 0.4–3.8 |
| >=172 | 17 | 17 | 50 | 1.9 | 0.6–5.6 |
| | | | | Trend test $P = 0.19$ | |
| BMI ($kg\,m^{-2}$) | | | | | |
| <=21.4 | 15 | 4 | 21 | 1.0 | |
| 21.5–25.4 | 16 | 15 | 48 | 3.5 | 1.0–13.0 |
| 25.5–28.4 | 16 | 14 | 47 | 3.3 | 0.9–12.2 |
| >=28.5 | 15 | 9 | 38 | 2.3 | 0.6–8.9 |
| | | | | Trend test $P = 0.42$ | |
| Occupational physical activity | | | | | |
| Sedentary | 15 | 11 | 42 | 1.0 | |
| Light | 21 | 9 | 30 | 0.6 | 0.2–1.8 |
| >=Medium | 29 | 22 | 43 | 1.0 | 0.4–2.7 |
| | | | | Trend test $P = 0.77$ | |
| Smoking | | | | | |
| No | 29 | 20 | 41 | 1.0 | |
| Yes | 33 | 22 | 40 | 1.0 | 0.4–2.1 |
| Drinking | | | | | |
| No | 21 | 16 | 43 | 1.0 | |
| Yes | 41 | 26 | 39 | 0.8 | 0.4–1.9 |
| Parity | | | | | |
| None | 8 | 6 | 43 | 1.0 | |
| 1–2 | 26 | 18 | 41 | 0.9 | 0.3–3.1 |
| 3+ | 27 | 17 | 39 | 0.8 | 0.2–2.8 |
| | | | | Trend test $P = 0.76$ | |
| Marital status | | | | | |
| Single | 4 | 6 | 60 | 1.0 | |
| Married | 47 | 30 | 39 | 0.4 | 0.1–1.6 |
| Widowed/divorced | 10 | 5 | 33 | 0.3 | 0.1–1.8 |

first-degree relatives with cancer had an odds ratio of 2.9 (95% CI 1.0–8.3) for p53 overexpression in comparison with those without family history of cancer (trend test, $P = 0.11$) (Table II). Patients who had a positive family history of cancer were further examined according to the tumour site in the first-degree relative. However, the data were too sparse to reveal any obvious trends.

### *Weight, height and body mass index (BMI) and p53 overexpression*

Increased body weight was associated with p53 overexpression. The odds ratio for p53 overexpression was 3.4 (1.0–11.9) for those at the highest quartile, compared with individuals at the lowest quartile (trend test, $P = 0.06$). No significant relationship between height and p53 overexpression is suggested by the data (Table III). No evidence of associations were found between p53 overexpression and smoking, drinking, occupational physical activity and parity (Table III).

### Discussion

There are conflicting reports as to whether accumulation of p53 protein as measured by immunohistochemical staining always equates the actual frequency of TP53 genomic mutations (Fisher *et al.*, 1994). Rodrigues *et al.* (1990) found that overexpression of p53 in colorectal cancer cell lines is synonymous with mutation, but some mutations could not be detected by IHC analysis. Cunningham *et al.* (1992) compared molecular and IHC techniques in colorectal cancer, and indicated that loss of heterozygosity of chromosome 17p by tumour cells correlated well with positive labelling for anti-p53 antibody. Our data (Dalbagni *et al.*, 1993; Cordon-Cardo *et al.*, 1994) suggested that the accuracy of detecting TP53 mutation by IHC was 90.3% in human bladder cancer. Identification of p53 nuclear accumulation by IHC has also been reported to correlate well with TP53 mutations, as determined by DNA sequencing analysis in breast (Jacquemier *et al.*, 1994), lung (Vahakangas *et al.*, 1992), and ovarian (Marks *et al.*, 1991) cancers.

Our study of colorectal cancer is disadvantaged by the fact that information on diet, the major risk factor, was unavailable because the study was retrospective, involving abstraction of data from the medical record and analysis of banked tissue specimens. The small sample size limits our ability to detect associations. But at present biomarker studies are difficult and expensive to conduct, and in context our study has a larger sample size than most other published molecular epidemiology studies (Field *et al.*, 1991; Holstein *et al.*, 1991; Kondo *et al.*, 1992; Suzuki *et al.*, 1992; Spruck *et al.*, 1993). Information abstracted from medical charges may be less reliable than from conventional interview studies. However, since the information on risk factors was collected by surgeons or physicians who interview patients using a standard admission history form, the potential for misclassification may be similar to conventional interview or questionnaire studies.

Family history of cancer is known to be associated with increased risk of colorectal cancer (Neugut *et al.*, 1993). In this study, p53-positive tumours occur 2.9 times more frequently in patients with two or more first-degree relatives with cancer. After controlling for body weight, the odds ratio was 3.0 (1.0–9.2). Our findings indicate that p53 overexpression may be related to genetic predisposition in colorectal cancer. It is possible that germline mutations may be present in the TP53 gene in some high-risk families, and that this is responsible for the association observed in our study.

Increased body weight has also been identified as a possible risk factor (Kreger *et al.*, 1992; Lee and Paffenbarger Jr., 1992). We found that the odds ratio of p53 overexpression was 3.4 for individuals at the highest quartile of body weight compared with those at the lowest quartile. The odds ratio was 3.1 (0.9–11.4) when adjusting for first-degree family members with cancer. The mechanism for the relationship between body weight and the risk of colorectal cancer is obscure. Increased body weight may be related to increased fat or caloric intake, decreased physical activity and metabolic and hormonal changes owing to increased body fat (Neugut *et al.*, 1993). Body weight correlates to dietary fat intake. In animals, high intake of dietary fat stimulates the secretion of bile and fatty acids. Increased concentration of bile and fatty acids in the colon may damage the surface epithelium of the colon, which in turn stimulates the replication of colonic epithelial cells (Lee and Paffenbarger Jr., 1992). Since a high proportion of transition-type point mutations of the TP53 gene have been found in human colon cancers, the association between increased body weight and p53 overexpression may, in part, be due to those endogenous determinants involved in carcinogenesis (Harris, 1993; Renault *et al.*, 1993).

We assessed the relationship between occupational physical activity and p53 overexpression and found no association. Since the occupational information obtained from the medical charts was the last occupation or the occupation at admission, our data may not accurately reflect lifetime occupational activity. We observed no apparent association between p53 overexpression and cigarette smoking in colorectal cancer in contrast to our earlier study of bladder cancer (Zhang *et al.*, 1994*a*).

We have shown that the odds ratio relating risk factors to the presence of a biological marker is an appropriate measure for characterising the degree of aetiological heterogeneity between the disease groupings defined by the biomarker (Begg and Zhang, 1994). In this study, we found that in patients with two or more first-degree members with cancer and those with high body weights had higher prevalence of p53 overexpression, which indicates that p53 overexpression may be related to genetic predisposition to colorectal cancer, and p53-positive and -negative colorectal cancers may be controlled by different aetiological pathways.

**Acknowledgements**
This publication was made possible in part by Grant No. R01 ES-06718 (Dr Zuo-Feng Zhang) from the National Institute of Environmental Health Science and Grant No. R01 CA-47538 (Dr Carlos Cordon-Cardo) from the National Cancer Institute, National Institute of Health, Department of Health and Human Services, and by Career Development Award 93-20 (Dr Jose Guillem) from the American Cancer Society.

### References

AMERICAN CANCER SOCIETY. (1994). *Cancer Facts & Figures – 1994*. American Cancer Society: Atlanta.

BANKS L, MATLASHEWSKI G AND CRAWFORD L. (1986). Isolation of human-p53-specific monoclonal antibodies and their use in the studies of human p53 expression. *Eur. J. Biochem.*, **159**, 529–534.

BEGG CB AND ZHANG ZF. (1994). Statistical analysis of molecular epidemiology studies employing case-series. *Cancer Epidemiol. Biomarkers Prev.*, **3**, 173–175.

BRACHMAN DG, GRAVES D, VOKE E, BECKETT M, HARAD D, MONTAG A AND 4 OTHERS. (1992). Occurrence of p53 gene deletions and human papilloma virus infection in human head and neck cancer. *Cancer Research*, **52(17)**, 4832–4836.

BRASH DE, RUDOLPH JA, SIMON JA AND 5 OTHERS. (1991). A role for sunlight in skin cancer: UV-induced p53 mutations in squamous cell carcinoma. *Proc. Natl Acad. Sci. USA*, **88**, 10124–10128.

BRESLOW NE AND DAY NE. (1980). *Statistical Methods in Cancer Research*, Vol. 1, *The Analysis of Case-Control Studies* IARC Scientific Publications No. 32. Vol. 1, pp. 122–159. IARC: Lyon.

CORDON-CARDO C, FINSTAD CL, BANDER NH, OLD LJ AND MELAMED M. (1987). Immunoanatomic distribution of cystostructural and tissue-associated antigens in the human urinary tract. *Am. J. Pathol.*, **126**, 269–284.

CORDON-CARDO C, DALBAGNI G, SAEZ GT, OLIVA MR, ZHANG ZF, ROSAI J, REUTER VE AND PELLICER A. (1994). p53 mutations in human bladder cancer: genotypic versus phenotypic patterns. *Int. J. Cancer*, **56**, 347–353.

CUNNINGHAM J, LUST JA, SCHAID DJ, BREN GD, CARPENTER HA, RIZZA E, KOVACH JS AND THIBODEAU SN. (1992). Expression of p53 and 17p allelic loss in colorectal carcinoma. *Cancer Res.*, **52**, 1974–1980.

DALBAGNI G, PRESTI J, REUTER VE, ZHANG ZF, SARKIS A, FAIR WR AND CORDON-CARDO C. (1993). Molecular genetic alterations of chromosome 17 and p53 nuclear overexpression in human bladder cancer. *Diagnostic Mol. Pathol.*, **2(1)**, 4–13.

DARMON E, CLEARY KR AND WARGOVICH MJ. (1994). Immunohistochemical analysis of p53 overexpression in human colonic tumors. *Cancer Detection Prev.*, **18(3)**, 187–195.

FEARON ER AND VOGELSTEIN B. (1990). A genetic model for colorectal tumorigenesis. *Cell*, **61**, 759–767.

FIELD JK, SPANDIDOS DA, MALLIRI A, GOSNEY JR, YIAGNISIS M AND STELL PM. (1991). Elevated P53 expression correlates with a history of heavy smoking in squamous cell carcinoma of the head and neck. *Br. J. Cancer*, **64**, 573–577.

FINLAY CA, HINDS PW, TAN TH, ELIYAHU D, OREN M AND LEVINE AJ. (1988). Activating mutations for transformation by p53 produce a gene product that forms an hsc70-p53 complex with an altered half-life. *Mol. Cell. Biol.*, **8**, 531–539.

FISHER CJ, GILLETT CE, VOJTESEK B, BARNES DM AND MILLIS RR. (1994). Problems with p53 immunohistochemical staining: the effect of fixation and variation in the methods of evaluation. *Br. J. Cancer*, **69**, 26–31.

GIOVANNUCCI E, GOLDITZ GA, STAMPFER MJ, HUNTER D, ROSNER BA, WILLETT WC AND SPEIZER FE. (1994*a*). A prospective study of cigarette smoking and risk of colorectal adenoma and colorectal cancer in U.S. women. *J. Natl Cancer Inst.*, **86(3)**, 192–199.

GIOVANNUCCI E, RIMM EB, STAMPFER MJ, GOLDITZ GA, ASCHERIO A, KEARNEY J AND WILLETT WC. (1994*b*). A prospective study of cigarette smoking and risk of colorectal adenoma and colorectal cancer in US men. *J. Natl Cancer Inst.*, **86(3)**, 183–191.

HARRIS CC. (1993). p53: at the crossroads of molecular carcinogenesis and risk assessment. *Science*, **262**, 1980–1981.

HARRIS CC AND HOLLSTEIN MC. (1993). Clinical implications of the p53 tumor-suppressor gene. *N. Engl. J. Med.*, **329**, 1318–1327.

HOLLSTEIN MC, PERI L, MANDARD AM, WELSH JA, MONTESANO R, METCALF RA, BAK M AND HARRIS CC. (1991). Genetic analysis of human esophageal tumors from two high incidence geographic areas: frequent p53 base stubstitutions and absence of ras mutations. *Cancer Res.*, **51**, 4102–4106.

HOLLSTEIN MC, WILD CP, BLEICHER F, CHUTIMATAEWIN S, HARRIS CC, SRIVATANAKUL P AND MONTESANO R. (1993). p53 mutations and alfatoxin B1 exposure in hepatocellular carcinoma patients from Thailand. *Int. J. Cancer*, **53**, 51–55.

HOWE GR, BENITO E, CASTELLETO R, CORNEE J, ESTEVE J, GALLAGHER RP, ISCOVICH JM, DENG-AO J, KAAKS R, KUNE GA, KUNE S, L'ABBE KA, LEE HP, LEE M, MILLER AB, PETERS RK, POTTER JD, RIBOLI E, SLATTERY ML, TRICHOPOULOS D, TUYNS A, TZONOU A, WHITTEMORE AS, WU-WILLIAMS AH AND SHU Z. (1992). Dietary intake of fiber and decreased risk of cancers of the colon and rectum: evidence from the combined analysis of 13 case control studies. *J. Natl Cancer Inst.*, **84**, 1887–1896.

HSU IC, TOKIWA T, BENNETT W, METCALF RA, WELSH JA, SUN T AND HARRIS CC. (1993). p53 gene mutation and integrated hepatitis B viral DNA sequences in human liver cancer cell lines. *Carcinogenesis*, **14**, 987–992.

JACQUEMIER J, MOLES JP, PENAULT-LLORCA F, ADELAIDE J, TORRENTE M, VIENS P, BIRNBAUM D AND THEILLET C. (1994). p53 immunohistochemical analysis in breast cancer with four monoclonal antibodies: comparison of staining and PCR-SSCP results. *Br. J. Cancer*, **69**, 846–852.

KASTAN MB, ONYKEWERE O, SIDRANSKY D, VOGELSTEIN B AND CRAIG RW. (1991). Participation of p53 protein in the cellular response to DNA damage. *Cancer Res.*, **51**, 6304–6311.

KONDO K, UMEMOTO A, AKIMOTO S, UYAMA T, HAYASHI K, OHNISHI Y. AND MONDEN Y. (1992). Mutations in the P53 tumour suppressor gene in primary lung cancer in Japan. *Biochem. Biophys. Res. Commun.*, **183**, 1139–1146.

KREGER BE, ANDERSON KM, SCHATZKIN A AND SPLANKSKY GA. (1992). Serum cholesterol level, body mass index, and the risk of colon cancer: the Framingham study. *Cancer*, **70**, 1038–1043.

LAVECCHIA C AND FRANCESCHI S. (1991). Reproductive factors and colorectal cancer. *Cancer Causes Control*, **2**, 193–200.

LEE I-M AND PAFFENBARGER JR RS. (1992). Quetelet's index and risk of colon cancer in college alumni. *J. Natl Cancer Inst.*, **84**, 1326–1331.

LEVINE AJ, CHANG PA, DITTMER AS, WU M AND WELSH D. (1994). The 1993 Walter Hubert Lecture: the role of the p53 tumour-suppressor gene in tumorigenesis. *Br. J. Cancer*, **69**, 409–416.

LONGNECKER MP, ORZA MJ, ADAMS ME, VIOQUE J AND CHALMERS TC. (1990). A meta-analysis of alcoholic beverage consumption in relation to risk of colorectal cancer. *Cancer Causes Control*, **1**, 59–68.

MARKS JR, DAVIDOFF AM, KERNS BJ, HUMPHREY PA, PENCE JC, DODGE RK, CLARKE-PEARSON DL AND IGLEHART JD. (1991). Overexpression and mutation of p53 in epithelial ovarian cancer. *Cancer Res.*, **51**, 2979–2984.

MILLER CW, SIMON K, ASLO A, KOK K, YOKOTA J, BUYS CH, TERADA M AND KOEFFLER HP. (1992). p53 mutations in human lung tumors. *Cancer Res.*, **52**, 1695–1698.

NEUGUT AI, JACOBSON JS AND DEVIVO I. (1993). Epidemiology of colorectal adenomatous polyps. *Cancer Epidemiol. Biomarkers Prev.*, **2**, 159–176.

PARKIN DM, PISANI P AND FERLAY J. (1993). Estimate of the world incidence of eighteen major cancers in 1985. *Int. J. Cancer*, **54**, 594–606.

PELTOMAKI P, AALTONEN LA, SISTONEN P, PYLKKANEN L, MECKLIN J-P, JARVINEN H, GREEN JS, JASS JR, WEBER JL, LEACH FS, PETERSEN GM, HAMILTON SR, DE LA CHAPELLE A AND VOGELSTEIN B. (1993). Genetic mapping of a locus predisposing to human colorectal cancer. *Science*, **260**, 810–812.

PISANI P, PARKIN DM AND FERLAY J. (1993). Estimates of the world mortality from eighteen major cancers in 1985. Implication and projections of future burden. *Int. J. Cancer*, **55**, 891–903.

POTTER JD, SLATTERY ML, BOSTICK RM AND GAPSTUR SM. (1993). Colon cancer: a review of the epidemiology. *Epidemiol. Rev.*, **15 (2)**, 499–545.

PRENTICE RL AND SHEPPARD L. (1990). Dietary fat and cancer: consistency of the epidemiological data, and disease prevention that may follow from a practical reduction in fat consumption. *Cancer Causes Control*, **1**, 81–97.

RENAULT B, VAN DEN BROEK M, FODDE R, WIJNEN J, PELLEGATA NS, AMADORI D, KHAN PM AND RANZANI GN. (1993). Base transitions are the most frequent genetic changes at P53 in gastric cancer. *Cancer Res.*, **53**, 2614–2617.

RODRIGUES NR, ROWAN A, SMITH ME, KERR IB, BODMER WF, GANNON JV AND LANE DP. (1990). p53 mutations in colorectal cancer. *Proc. Natl Acad. Sci. USA*, **87**, 7555–7559.

SPRUCK III CH, RIDEOUT III WM, OLUMI AF, OHNESEIT PF, YANG AS, TSAI YY, NICHOLS PW, HORN T, HERMANN GG, STEVEN K, ROSS RK, YU MC AND JONES PA. (1993). Distinct pattern of p53 mutations in bladder cancer: relationship to tobacco usage. *Cancer Res.*, **53**, 1162–1166.

SUZUKI H, TAKAHASHI T, KUROISHI T, SUYAMA M, ARIYOSHI Y AND UEDA R. (1992). p53 mutations in non-small cell lung cancer in Japan: association between mutations and smoking. *Cancer Res.*, **52**, 734–736.

US EMPLOYMENT SERVICE DEPARTMENT OF LABOR, BUREAU OF EMPLOYMENT SECURITY. (1956). *Estimates of Worker Trait Requirements for 4,000 jobs as Defined in the Dictionary of Occupational Titles (an alphabetical index)*. USGPO: Washington, DC.

VAHAKANGAS KH, SAMET JM, METCALF RA, WELSH JA, BENNETT WP, LANE DP AND HARRIS CC. (1992). Mutations of p53 and ras genes in radon-associated lung cancer from uranium miners. *Lancet*, **339**, 576–580.

VENA JE, GRAHAM S, ZIELENZNY M, SWANSON MK, BARNES RE AND NOLAN J. (1985). Lifetime occupational exercise and colon cancer. *Am. J. Epidemiol.*, **122(3)**, 357–365.

VOGELSTEIN B, FEARON ER, HAMILTON SR, KERN SE AND 6 OTHERS. (1988). Genetic alterations during colorectal tumor development. *N. Engl. J. Med.*, **319**, 525.

WYLLIE AH. (1993). Apoptosis (The 1992 Frank Rose Memorial Lecture). *Br. J. Cancer*, **67**, 205–208.

ZENG ZS AND ZHANG ZF. (1993). Cholecystectomy and colorectal cancer in China. *Surgical Oncol.*, **2**, 311–319.

ZENG ZS, SARKIS AS, ZHANG ZF, KLIMSTRA DS, CHARYTONOWICZ E, GUILLEM JG, CORDON-CARDO C AND COHEN AM. (1994). p53 nuclear overexpression: an independent predictor of survival in lymph node positive colorectal cancer patients. *J. Clin. Oncol.*, **12** (10), 2043–2050.

ZHANG ZF, SARKIS AS, CORDON-CARDO C, DALBAGNI G, MELAMED J, APRIKIAN A, POLLACK D, SHEINFELD J, HERR HW, FAIR WR, REUTER VE AND BEGG C. (1994*a*). Tobacco smoking, occupation, and p53 nuclear overexpression in early stage bladder cancer. *Cancer Epidemiol. Biomarkers Prev.*, **3**, 19–24.

ZHANG ZF, APRIKIAN A, SARKIS AS, ZENG ZS, POLLACK D, CORDON-CARDO C, FAIR WR AND BEGG CB. (1994*b*). Factors associated with p53 accumulation in prostatic adenocarcinoma. *Int. J. Oncol.*, **4**, 897–901.